# Comparison of plant growth and remediation potential of pyrochar and thermal desorption for crude oil-contaminated soils

**DOI:** 10.1038/s41598-021-82243-y

**Published:** 2021-02-02

**Authors:** Noshin Ilyas, Uzma Shoukat, Maimona Saeed, Nosheen Akhtar, Humaira Yasmin, Wajiha Khan, Sumera Iqbal

**Affiliations:** 1 Department of Botany, PMAS-Arid Agriculture University, Rawalpindi, Pakistan; 2 Department of Biosciences, COMSATS University Islamabad (CUI), Islamabad, Pakistan; 3 Department of Biotechnology, COMSATS University Islamabad, Abbottabad Campus, Pakistan; 4 Department of Botany, Lahore College for Women University, Lahore, Pakistan

**Keywords:** Plant sciences, Environmental sciences

## Abstract

Crude oil contamination is a serious environmental threat for soil and plants growing in it. This study provides the first experimental evidence for comparison of the efficacy of pyrochar (slow pyrolysis biochar), thermal desorption and their combined application for degradation of crude oil contaminated soil (0%, 10%, and 20%), and growth of lettuce under glasshouse conditions. Pyrochar was produced by pyrolysis of sawdust at 350 °C, whereas thermal desorption was done by soil pyrolysis at 500 °C. Soil incubations were done for 120 days. The results of soil analysis showed that the crude oil degradation efficiency for the combined application was highest (40%), whereas pyrochar and thermal desorption was 25% and 19.6%, respectively. The maximum degradation products of crude oil were manifested by the detection of low molecular weight hydrocarbons (ranged between 173 and 422) in the soil with combined application treatment using Gas Chromatography-Mass Spectrometry (GC–MS) analysis. Crude oil contamination significantly reduced the germination and growth of the lettuce plants. Similarly, the combined application also improved plant growth by an increase of 24% in germination percentage, 35.5% in seedling vigor index, and 27% in promptness index under 20% crude oil contamination. Remediation caused a significant increase in fresh and dry biomass (40%), leaf area (30%), total chlorophyll (21%), water potential (23.6%), osmotic potential (27%), and membrane stability index (40%). Moreover, there was an increase in the contents of proline (32%), total amino acids (29%), soluble sugars (37%), proteins (27%), and antioxidant enzymes such as superoxide dismutase (19%), catalase (33%) and peroxidase (38%). This study confirmed the efficacy of pyrochar (slow pyrolysis biochar), thermal desorption, and their combined application for crude oil decontamination of soil at laboratory scale and also in improving soil usability by improved germination and growth of lettuce.

## Introduction

The release of petroleum oil in soil and water, due to various human activities, is posing severe threats to our environment. Petroleum hydrocarbons are considered very hazardous to living organisms due to their toxicity, mutagenicity, and carcinogenicity^1^. Contamination of soil with oil results in serious depression of growth of most plants, primarily due to its effects on the physical and chemical properties of soil and soil water relations^2^.

Crude oil affects the environment by changing the essential elements of habitat. To overcome this problem, different soil remediation technologies have been developed such as soil washing with surfactants, biological treatment, thermal treatment, air stripping etc.^3^. Thermal desorption treatment has been considered one of the very promising technique due to its possible applications to a wide range of organic contaminants and some heavy metals (Hg), converting them to volatile forms. Nowadays, pyrolysis is being done during which the contaminated soil is heated between 350 and 500 °C, which also reduces heat requirement from 40 to 60% as compared to other conventional heating methods^4^. According to Ren et al.^5^, 10 min of pyrolysis at 250 °C of soil containing diesel reduced total petroleum hydrocarbons from 6272 to 359 mg/kg. However, it affects soil porosity and properties, which are not easy to be recovered^6^.

Pyrolysis through the carbonization of biomass at processing temperatures of above 300 °C under oxygen-free conditions, produces pyrochar. Pyrochar is a new and effective soil amendment which can maintain soil nutrient and enhance carbon sequestration. Pyrochar also shows great potential for water conservation and water use efficiency improvement under greenhouse cultivation. Pyrochar has a high potential for carbon sequestration and acts as activated carbon and can be effectively used in soil bioremediation processes^7^. Moreover, pyrochar has improved physical and chemical properties and has the potential for soil amendment, carbon sequestration, pollution remediation, and bioenergy production^8^.

Lettuce (*Lactuca sativa* L.) plant is very sensitive towards soil hydrocarbons^9^. Lettuce seed and growth bioassays can be effectively used for the evaluation of soil toxicity due to contamination^10^. Seed germination assays, particularly of lettuce, can predict phytotoxicity of contaminated soils^11^. Marti et al.^12^ used lettuce growth parameters as an indicator of the toxicity of soil samples.

Thermal desorption is a very effective technique for decontamination of oil-polluted soils but thermal treatments lead to changes in soil fertility and water levels which can limit its use for vegetation^4^. In contrast, biochar has less remediation potential but can change the soil physicochemical properties^13^, improve the water holding capacities of the soil^14^ eventually resulting in increased soil fertility^15^; with potential beneficial effects on crop productivity, plant establishment, and growth^13^. Hence, the integration of thermal desorption with the biochar amendment can not only lead to enhanced remediation of crude oil but can also prove effective for better plant growth. Currently, to the best of our knowledge, there is no published record on the comparison of pyrochar and thermal desorption, alone or in the combined application. In this study, we have investigated the remediation of crude oil contaminated soil through pyrochar addition and thermal desorption of crude oil-contaminated soil. Treated soils were used to study the germination of lettuce and its morphological, physiological, and biochemical responses were used as indicators for soil toxicity.

## Results

The present research was designed to observe the remediation potential of a new combined application technique i.e. pyrochar (slow pyrolysis biochar); thermal desorption alone and their combined application in the crude oil-polluted soil. We found that this new combined application was effective not only in remediating the soil but also neutralized the toxic nature of crude oil.

### Soil analysis

Crude oil contamination negatively affected soil properties by causing a decrease in soil pH (3% and 7% at 10% and 20% contamination), EC (10% and 28% at 10% and 20% contamination), and soil moisture content (27.6% and 41.5% at 10% and 20% contamination). Results showed a significant reduction in available nutrients. The nitrogen, phosphorous, and potassium showed a maximum reduction by 29 and 40%, 7%, and 15%, and to trace quantities at 10% and 20% contamination, respectively as compared to the non-contaminated soil. However, organic carbon improved significantly by two and three times, at 10% and 20% contamination. Pyrochar improved soil characteristics not only under control conditions but also under oil contamination. Thermal desorption had little effect on soil properties. Soil amendments with pyrochar and thermal desorption improved the properties of soil.

The most promising results were shown by the combined application resulting in 30% and 27.3% increase in soil moisture content at 10% and 20% contamination, 40% and 23% increase in nitrogen contents at 10% and 20% contamination, 10–3% in phosphorous content and from trace level to detectable amounts in potassium for 10% and 20% contaminated soil, respectively (Supplementary Table 1).

### Crude oil degradation

The effect of pyrochar and thermal desorption on the degradation of hydrocarbons was significant (p ≤ 0.5). An inverse relationship exists between the rate of hydrocarbon degradation and the level of oil contamination as hydrocarbon degradation decreased with an increase in the oil contamination. Treatment of soil with a combined application of pyrochar and thermal desorption showed more promising results of 40.1% degradation of hydrocarbons which is 30 folds greater as compared to control one at 20% level of crude oil. The addition of pyrochar showed 25% hydrocarbon degradation which is 13.8 times more compared to the control one. While 19.7% of degradation of hydrocarbons was recorded with thermal desorption as compared to control one. The degradation order of effectiveness is combined application treatment > pyrochar > thermal desorption > control (Table 1). So, a combined application of pyrochar and thermal desorption is more effective and is suggested as possible remediation of hydrocarbon contamination. An inverse relationship exists between the rate of hydrocarbon degradation and the level of oil contamination as hydrocarbon degradation decreased with an increase in the oil contamination. (Table 1).

**Table 1:** Degradation rate of hydrocarbons in control, pyrochar, and thermal remediated soil.

Treatments	Degradation rate (%)	
	10% oil contamination	20% oil contamination
Control	11.25 ± 0.5d	8.5 ± 0.01a
Pyrochar	25.09 ± 0.02b	16.12 ± 0.1b
Thermal desorption	19.65 ± 0.05c	14.75 ± 0.5c
Pyrochar + Thermal desorption	40.15 ± 0.1a	32.31 ± 0.02a

This data displays the means and standard deviation (n = 3). Different letters show significant differences (p < 0.05) following the order a > b > c > d.

### GC–MS analysis

GC–MS analysis of soil samples for the identification of compounds being generated after the degradation of crude oil. The results suggested that crude oil was degraded in soil by different treatments after the 120 days’ trial. The mass spectrometer identified the hydrocarbon constituents using the NIST Library. We found a range of compounds (aliphatic and aromatic) with different molecular weights (173–422). The data generated the total ion chromatograms, which revealed that the transformations of parent compounds (crude oil) are associated with the production of metabolites. The metabolites generated during the degradation of a quaternary mixture of PAHs provided useful information about the biodegradation process. GC–MS analysis showed that high molecular weight compounds were degraded, and the peak area indicated the presence of hydrocarbons in soil (Table 2). After 120 days’ trial, the products of soil treated with combined application treatment were Dodecane, Undecane 3,8 dimethyl, Dodecane 2,6,11, Trimethyl, *n*-Hexadeconic acid, Hexadecane, and Flouranthene. These possibly originated from the synergistic effect of pyrochar and thermal desorption. Whereas, pyrochar and thermal desorption resulted in the production of a lesser number of compounds (9H-Flourine, 9-methylene, 2-Bromotetradecane, Hexdecane, Pyrene were produced by pyrochar whereas thermal desorption produced Heptadecane, Heptadecane 6,10 tetramethyl, 7,9 Di-tert-butyl-1-oxaspiro (4,5), Phthalic acid bis (7-methyl octyl) ester). Overall, the GC–MS analysis showed that combined application treatment degraded PAH into more number of lesser molecular weight compounds and thus more effective in decontamination.

**Table 2:** The end products and metabolites of crude oil produced by degradation by pyrochar, thermal sorption, their combined application and contaminated soil.

Treatments	Retention time	Peak area(%)	Compound	Molecular formula	M.W
Control	15.3	71,785 × 10^3^	Butyldodecyl ester	C_24_H_38_O_4_	390.6
	19.11	121,541 × 10^3^	Butyltetradecyl ester	C_26_H_42_O_4_	384.59
	23.18	61,452 × 10^3^	Triacontane	C_30_H_62_	422.81
Pyrochar	11.13	75,694 × 10^3^	9H-Flourine, 9-methylene	C_14_H_30_	178
	14.24	45,861 × 10^3^	2-Bromotetradecane	C_14_H_29_Br	276
	17.11	65,319 × 10^3^	Hexdecane	C_16_H_34_	226
	22.44	51,823 × 10^3^	Pyrene	C_16_H_10_	202
	26.42	35,481 × 10^3^	Tridecane 1-iodo	C_13_H_29_I	310
Thermal desorption	12.15	54,291 × 10^3^	Heptadecane	C_17_H_36_	240.48
	16.32	47,523 × 10^3^	Heptadecane 6,10 tetrmethyl	C_21_H_44_	296.16
	23.45	42,156 × 10^3^	7,9 Di-tert-butyl-1-oxaspiro(4,5)	C_17_H_24_O_3_	276
	26.56	34,612 × 10^3^	Phthalic acid bis(7-methyloctyl) ester	C_26_H_42_O_4_	418
Combined application	10.53	58,432 × 10^3^	Dodecane	C_12_H_26_	170.33
	13.4`2	42,713 × 10^3^	Undecane 3,8 dimethyl	C_13_H_28_	200.36
	19.11	52,341 × 10^3^	Dodecane 2,6,11, trimethyl	C_15_H_32_	212
	19.45	34,267 × 10^3^	n-Hexadeconic acid	C_16_H_32_O_2_	256
	23.16	54,243 × 10^3^	Hexadecane	C_16_H_34_	226
	26.54	45,261 × 10^3^	Flouranthene	C_16_H_10_	202

### Plant germination

The effect of soil remediation through pyrochar, thermal desorption, and their combined application had shown a significant effect (p ≤ 0.05) on the germination of two lettuce varieties. Crude oil stress decreased the germination percentage up to 29.7% and 39% in 10% and 20% contaminated soil as compared to the control. Application of combined application (pyrochar and thermal desorption) resulted in 28.5% and 21.8% increase in germination percentage of both lettuce varieties as compared with respective control stress of 10% and 20% contaminated soil. There was a 24% and 17.5% increase in germination percentage by pyrochar and 19% and 12.3% by thermal desorption 10% and 20% contaminated soil. The seedling vigor index also increased by 35.5% and 28.7% by pyrochar and thermal desorption remediation in 20% contaminated soil. Similar trends were observed in the promptness index (Supplementary Fig. 1).

### Plant morphology

**Figure 1:**
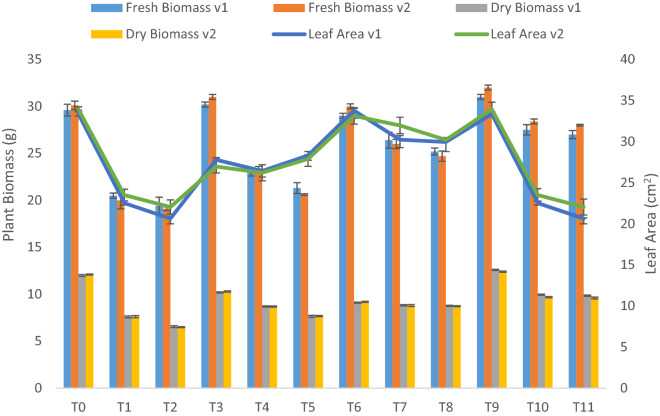
Fresh and dry biomass and leaf area of two lettuce varieties growing in crude oil contaminated, pyrochar, thermal desorption, and their combined application remediated soil. Detail of treatments: Where, T0 = control soil, T1 = 10% crude oil contaminated soil, T2 = 20% crude oil contaminated soil, T3 = Pyrochar treated control soil, T4 = Pyrochar + 10% crude oil contaminated soil, T5 = Pyrochar + 20% crude oil contaminated soil, T6 = Thermal desorption control soil, T7 = Thermal desorption + 10% crude oil contaminated soil, T8 = Thermal desorption + 20% crude oil contaminated soil, T9 = Pyrochar + Thermal desorption control soil, T10 = Pyrochar + Thermal desorption + 10% crude oil contaminated soil, T11 = Pyrochar + Thermal desorption + 20% crude oil contaminated. soil V1 = Iceberg, V2 = Boston. This data displays the means and standard deviation (n = 3). Different letters show significant differences (p < 0.05).

Plant fresh and dry biomass (Fig. 1) showed a considerable decrease (30% and 40% in 10% and 20% contaminated soil) and leaf area (25% and 30% in 10% and 20% contaminated soil). We found that combined application treatment had the most pronounced results by showing a maximum increase of plant biomass up to 40% (10% contamination) and 22% (20% contamination), respectively. Soil amendment with pyrochar helped to mitigate the effect of crude oil and increased by 30% and 38% biomass at 10% and 20% contaminated soil. Whereas, thermal desorption improved growth by 12% (10% contamination) and 10% (20% contamination). Both varieties performed well in response to the degradation potential of pyrochar, thermal desorption, and their combined application (Fig. 1).

### Plant physiology

**Figure 2:**
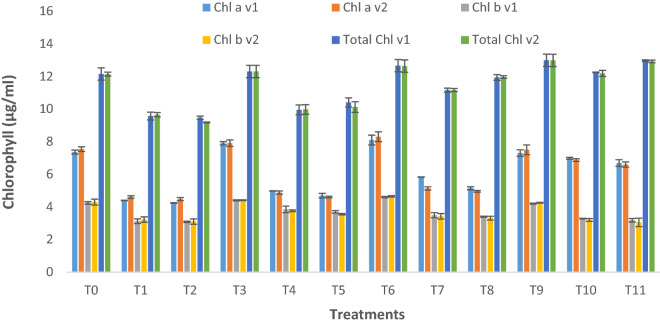
Chlorophyll a, chlorophyll b, and total chlorophyll contents of two lettuce varieties growing in crude oil contaminated, pyrochar, thermal desorption, and their combined application remediated soil. This data displays the means and standard deviation (n = 3). Different letters show significant differences (p < 0.05). The detail of treatments is the same as in Fig. 1.

There was a decrease in Chlorophyll a (40% and 45% decrease at 10–20% contamination, respectively), Chlorophyll b (26% and 30% decrease at 10–20% contamination, respectively), and total chlorophyll (20% and 25% decrease at 10–20% contamination, respectively) contents (Fig. 2), with an increase in the concentration of crude oil compared to non-polluted soils condition. Combined application treatment (Pyrochar and thermal desorption) improved the plant growth and total chlorophyll contents by 21 and 17% at 10–20% contamination respectively, as compared to the stressed plants.

**Figure 3:**
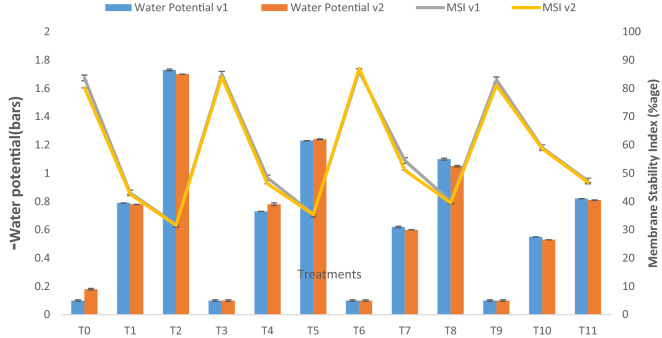
Water potential and membrane stability index of two lettuce varieties growing in crude oil contaminated, pyrochar, thermal desorption, and their combined application remediated soil. This data displays the means and standard deviation (n = 3). Different letters show significant differences (p < 0.05). The detail of treatments is the same as in Fig. 1.

Similarly, it was observed that the presence of 10% and 20% crude oil contamination decreased water potential by one and one and a half fold whereas combined application treatment improved water potential by 33.6% and 26.9% at respective low (10%) and high (20%) crude oil concentration (Fig. 3). In our observations, pyrochar increased the water potential of the leaf by 10% and 21% at 10–20% contamination, respectively. Whereas thermal desorption improved water potential by at 24% and 20% at 10–20% contamination, respectively Pyrolysis treatment remediated the soil and improved plant parameters.

Under 10% and 20% contamination crude oil contamination, the membrane stability index (MSI) decreased to 38.4% and 40.5% as compared to the control. Pyrochar addition resulted in a 32% and 21.6% increase in MSI in plants under both levels of crude oil. Thermal desorption showed an increase of 13% and 9.5% as compared to the stressed plants, whereas pyrochar showed a respective increase of 18% and 11%, as compared to respective control. A similar response was shown by both varieties.

### Plant biochemistry

**Figure 4:**
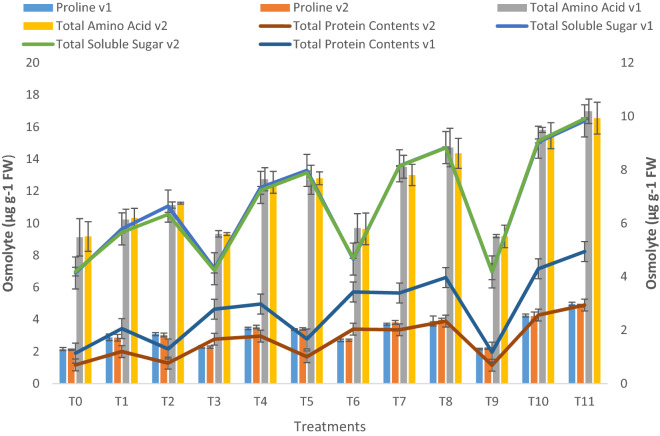
Proline contents, total amino acids, soluble sugar, and protein contents of two lettuce varieties growing in crude oil contaminated, pyrochar, thermal desorption, and their combined application remediated soil. This data displays the means and standard deviation (n = 3). Different letters show significant differences (p < 0.05). The detail of treatments is the same as in Fig. 1.

Oil contamination stress caused an increase in proline (33% and 43% decrease at 10–20% contamination, respectively), the free amino acid (12% and 22% decrease at 10–20% contamination, respectively), total protein (18% and 25% decrease at 10–20% contamination, respectively), and soluble sugar content (39% and 60% decrease at 10–20% contamination, respectively) in both verities of lettuce (Fig. 4).

Combined application treatment (pyrochar and thermal desorption) increased proline contents by 25% and 32% under 10% and 20% contamination, respectively, pyrochar showed less increase in proline contents (6% under control, 18% at 10% contamination and 9% at 20% contamination), whereas thermal desorption resulted in (25% increase under control conditions, 30% at 10% contamination and 28% at 10% contamination., with respect to respective control).

A significant increase in total amino acid by combined application treatment was observed i.e. 29% and 16% with 10% and 20% contamination, respectively. Pyrochar increased total amino acid by 24% and 13% at 10–20% contamination, respectively, whereas thermal decontamination caused a 30% and 27% increase at 10–20% contamination, respectively.

The total soluble sugar concentration of lettuce plants was increased by the combined application by 37% and 22% at 10% and 20% contamination, respectively, however, pyrochar increased total soluble sugar by 26% and 20% at 10% and 20% contamination and thermal desorption increased 31 and 17% at 10% and 20% contamination, respectively.

Similarly, total protein contents increased by combined application treatment by 27% and 19% at 10% and 20% contamination, respectively. Pyrochar increased total protein by 20% and 17% at 10% and 20% contamination, respectively and thermal desorption increased and 22% and 18% at 10% and 20% contamination, respectively. Results were significant in both varieties.

### Plant antioxidants

**Figure 5:**
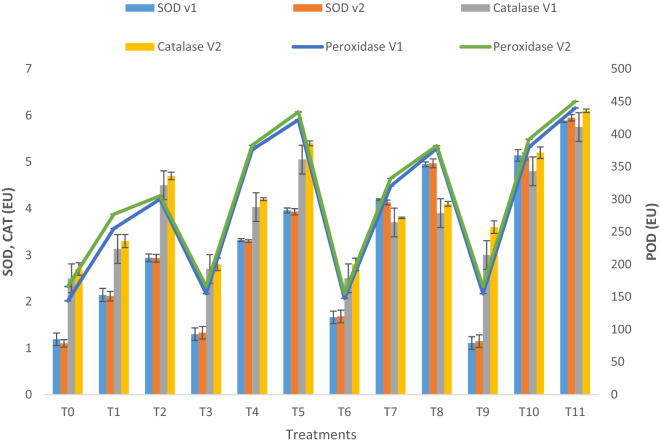
Antioxidant enzymes activity of two lettuce varieties growing in crude oil contaminated, pyrochar, thermal desorption, and their combined application remediated soil. This data displays the means and standard deviation (n = 3). Different letters show significant differences (p < 0.05). The detail of treatments is the same as in Fig. 1.

A notable rise in antioxidants was recorded in crude oil contaminated conditions (Fig. 5). Combined application treatment (Pyrochar and thermal desorption) increased superoxide dismutase activity by 140% and 100% in lettuce plants grown under 10% and 20% contamination, respectively. Pyrochar increased SOD by 55% and 34%, thermal desorption by 82% and 68% at 10% and 20% contamination, respectively. Combined application treatment (Pyrochar and thermal desorption) treatment significantly reduces the adverse effects of crude oil contamination by increasing catalase activity by 53% and 27% in plants grown under 10% and 20% contamination, respectively. The increase in catalase by pyrochar was 28% and 13% and thermal desorption by 185% and 10% at 10% and 20% crude oil contamination, respectively. Similarly, lettuce plants when treated with combined application treatment (Pyrochar and thermal desorption) showed pronounced peroxidase activity by 49% and 46% at 10% and 20% contamination, respectively. Pyrochar treatment showed a significant increase of 9% and 13% in treated plants at 10% and 20% crude oil contamination. Whereas, thermal desorption increased peroxidase activity by 25% and 20% at 10% and 20% crude oil contamination, respectively. Both varieties of lettuce responded to bioremediation.

### Heatmap responses of Pearson’s correlation coefficient (r)

**Figure 6:**
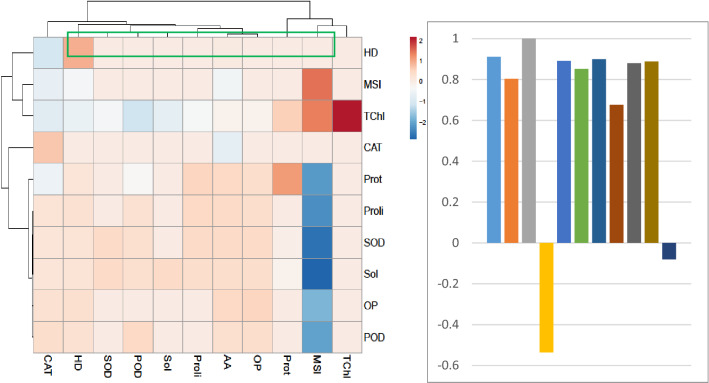
Heatmap responses of Pearson’s Correlation Coefficient (r) for the crude oil degradation, antioxidant enzymes, chlorophyll contents, osmolyte production of lettuce leaves growing in crude oil contaminated soil treated with pyrochar, thermal desorption, and their combined application. Whereas *TChl* Total Chlorophyll, *MSI* Membrane Stability Index, *CATL* Leaves catalase, *PODL* Leaves peroxidase, *SODL* Leaves superoxide dismutase, *AA* Amioacid, *Prol* Proline, *OP* Osmotic potential, *Sol* Soluble Sugars.

For heat map analysis the data of both lettuce seedlings under crude oil contamination stress were classified as hydrocarbon degradation, antioxidant enzymes, chlorophyll contents, osmolyte production, and each group showed positive correlations (Fig. 6). A comparative analysis of the factors related to hydrocarbon degradation (presented by green boxes) suggested that hydrocarbon degradation in contaminated soil had a positive correlation with amino acid, osmotic potential, soluble sugars, proline, MDA, SOD, POD, and CAT activities. While a negative correlation between total chlorophyll and MSI was observed. These results indicate that the degradation efficiency of combined application treatment (pyrochar and thermal desorption) for crude oil contamination resulted in better growth of lettuce plants.

## Discussions

Crude oil contamination affects the agriculture sector particularly due to soil toxicity and reduced plant growth. Crude oil causes environmental risks in the soil ecological system^16^, by inhibition of plant growth, damage to soil structure, disturbance of soil water quality, and so on^17^. The present research was designed to observe the remediation potential of a novel combined application treatment (pyrochar and thermal desorption) technique, thermal desorption alone, and pyrochar alone in the oil-polluted soil. We found that these methods are not only beneficial to remediate the soil but also capable to overcome the toxic nature of crude oil.

Pyrochar improved soil characteristics not only under control conditions (without any contamination) but also under oil contamination. Thermal desorption had little effect on soil properties. The most promising results were shown by the combined application treatment. Soil amendments with combined application treatment (pyrochar and thermal desorption) improved the properties of soil. In thermal desorption, the soil contaminants are desorbed, mobilized, and evaporated by the rise in temperature (from 100 to 600 °C). Besides, thermal desorption also promotes biodegradation but decreases soil fertility^18^. Relatively limited studies are available on the application of pyrochar on the remediation of soil contaminated with organic pollutants^19^. However, little is currently known about the effects of pyrochar combined application with thermal desorption technique on the remediation of petroleum-contaminated soil. However, it has been observed in the present study that this combined application treatment effectively improved soil characteristics both at 10% and 20% crude oil contamination. This may be possibly due to the synergistic action of pyrochar and thermal desorption under their combined application in pyrochar. This is mainly due to the greater absorptive capacity of pyrocahr’s greater surface area and microporous structure. Pyrochar enhances the nutrient content of soil for microbial populations and absorbed hydrophobic compounds more strongly. Improved microbial population stimulates the breakdown of absorbed hydrocarbons and used it as a source of carbon and energy. While thermal desorption accelerates the degradation of hydrocarbons. In thermal desorption, soil properties get changed and this type of soil is not supportive for the growth of plants. So in a combined application with pyrochar and thermal desorption, pyrochar improves the status of nutrients and holds hydrocarbons on its surface for degradation by microbes and thermal desorption and so this not only helps in degradation also enhances the nutrient status of the soil.

Treatment of soil with combined application treatment (pyrochar and thermal desorption) showed the most promising results in the degradation of crude oil. An inverse relationship exists between the rate of hydrocarbon degradation and the level of oil contamination as hydrocarbon degradation decreases with an increase in the oil contamination. This treatment degraded the crude oil contaminants in more members of low molecular weight compounds at both contamination levels (10% and 20%). In combined application treatment, both pyrochar and thermal desorption techniques acted synergistically and their combined application significantly improved the degradation potential. Thermal desorption accelerates the degradations whereas pyrochar has also shown the potential of oil degradation and promotes microbial degradation^20^. Banat^21^ also reported that organic compounds in crude oil could be metabolized by oil-degrading microbes which are stimulated by soil amendment. Bioremediation of crude oil contaminated soil by stimulating the activity of bacteria by the addition of poultry manure^22^.

Removal of majority n-alkanes happened by pyrochar indicates that it has the potential in the remediation of crude oil. The results are consistent with the standards set for effective remediation processes^23^. Pyrochar can increase the sorption of organic pollutants^24^. Kang et al.^18^ attributed the thermal sorption to degradation of PAH. So, the combined application of pyrochar and thermal desorption can be preferentially used for oil-contaminated soils for biotreatment. The pyrochar improves the soil nutrient status for microorganisms^25^ including organic carbons and binds hydrophobic organic compounds (HOCs), such as PAHs, more strongly. The thermal desorption technique is a very useful remediation technology because it is highly effective in decontaminating pollutants and organic chemicals. After thermal treatment, soil structure and properties were affected and the porosity of soil changes which is not easy to be recovered^6^. The synergism of pyrochar and thermal desorption resulted in enhanced degradation of n-alkanes at both low (10%) and high (20%) contamination levels.

Overall, crude oil contamination showed adverse effects on the growth of lettuce plants by reducing plant vigor, biomass, yellowing, and wilting of leaves. However, all soil treatments showed a significant reduction in crude oil toxicity and ultimately improvement in the growth of lettuce plants. A higher level of contamination (20%) had more drastic reductions as compared to low (10%) crude oil contamination). Application of combined application treatment (pyrochar and thermal desorption) results in enhanced seed germination, plant establishment, and growth; ultimately increasing crop productivity^13^. Pyrolysis of oil-contaminated soil results in higher exchange capacity, surface area, and direct nutrient additions and hence in better growth promotions^26^. The thermal desorption technique is a very useful remediation technology because it is highly effective in decontaminating pollutants and organic chemicals. After thermal treatment, soil structure and properties were affected and the porosity of soil changes which is not easy to be recovered^6^. No prior information is available about the response of plant germination to pyrochar application or its combined application with thermal desorption. However, our results are showing that this new technique of combined application treatment (pyrochar and thermal desorption) performed better than both pyrochar and thermal desorption techniques, alone, and thus its application can significantly improve plant growth and germination attributes.

Various studies have reported the role of these remediation techniques in the improvement of plant growth^27^ who also observed a decrease in biomass of plant under oil stress. Our results also relate with Laird et al*.*^28^*,* where fresh weight and dry weight increased because of the pyrochar amendment in soil. Thereby, pyrochar may reduce the leaching losses of nutrients by adding nutrients to the soil. Pyrolysis of soil was effective in soil remediation and soil biomass increased when compared with stress-imposed plants^18^. A combined application of both these treatments was most effective in the improvement of plant growth under crude oil contamination. This may be due to the reason that as it’s a combined application technique, and combined effect of pyrochar and thermal desorption can complement each other and thus prove to be more effective.

Chlorophyll a, b, and total chlorophyll contents decreased with an increase in the concentration of crude oil compared to non-polluted soil conditions. Baruah et al.^29^*,* have been reported a similar decline in chlorophyll content of plants. Combined application treatment (pyrochar and thermal desorption) improved plant growth and chlorophyll content. Pyrochar and pyrolysis did improve plant pigments, but their effects were less pronounced. The increase in chlorophyll content might be due to an increase in nutrient uptake by the plant and a decrease in crude oil uptake^30,31^. Our results correlate with Ali et al.^32^*,* who reported that crude oil adversely affects the plant-water-soil relationship, resulting in chlorosis. To maintain the pressure potential, the plant needs to reduce the osmotic potential^33^. Although the use of pyrochar as a soil amendment is anticipated to increase the soil nutrients and water use efficiency and thereby crop productivity^34^. Pyrochar treated soil had better nutrient concentrations and water contents and thus improved plant water relations and ultimately increased plant photosynthetic pigments.

Stress causes an increase in proline, free amino acid, total protein, and soluble sugar content in both verities of lettuce. Proline act as an osmotic stress protectant in response to environmental stress tolerance^35^. Under stressed conditions, proline plays a role in the stabilization of membranes, proteins, and other subcellular structures and protecting the cell by an increased level of reactive oxygen species. It helps to maintain the pH and turgor pressure of the cell^36^. Similar results were lined with Wang et al*.*^23^*.* A significant increase in total amino acid was observed i.e. 29% and 16% with 10 and 20% contamination, respectively. Amino acids act as osmoregulators in plants under stress conditions^34^. Under stress conditions, high soluble sugars regulate physiological roles such as photosynthesis, reserve mobilization, and exports while low sugar concentration promotes carbohydrate storage and senescence^37^. In present findings, though pyrochar and thermal pyrolysis treatment improved the proline, amino acids, and soluble sugar contents content under crude oil stress, the greatest increase was due to synergism of biochar and thermal desorption.

Antioxidants protect the cell from any damage caused by cytotoxic O_2_, by stopover its conversion to H_2_O_2_ and O_2_ in all the subcellular compartments. Combined application treatment (pyrochar and thermal desorption) treatment significantly improved plant antioxidant systems and helped plants to prevent damage by reactive oxygen species. Pyrochar treatment showed a significant increase of 9% and 13% in treated plants at 10% and 20% crude oil contamination. The remediation processes may increase the antioxidants by stimulating the intake of nitrogen and phosphorus, which interact with carbohydrates as non-enzymatic antioxidants^38^.

Correlation analysis showed that hydrocarbon degradation in contaminated soil is positively correlated with lettuce plant growth by regulating plant defense responses including antioxidant enzymes and osmolytes. This study establishes the relationship between the degradation potential of the new technique of combined application treatment (pyrochar and thermal desorption) with improved soil properties and ultimately in plant growth.

## Conclusion and future perspectives

The results of the present research have proven the bioremediation potential of a new combined application treatment (pyrochar and thermal desorption) technique, for crude oil contamination which also showed significant growth potential for the lettuce plants. It enhanced the morphological, physiological, and biochemical parameters of the plant. Pyrochar and thermal pyrolysis treatments, alone, had their limitations in terms of slow degradation rates and less efficiency in plant growth, respectively. However, combined application treatment application proved effective in both processes and hence can be used at a larger scale on soils where crude oil pollution is a serious problem particularly for agricultural production.

## Methodology

### Preparation of soil

Non-polluted soil samples were obtained from the experimental field area of PMAS-Agriculture University, Rawalpindi, which had no prior exposure to hydrocarbons. The soil was sieved and was divided into three parts, 1/3 of soil was kept clean and was taken as control, the second was Low Crude oil Contamination contaminated with 10% (w/w) and the third part was High Crude oil Contamination contaminated with 20% crude oil (w/w), respectively.

Pyrochar was produced by pyrolysis of sawdust and shavings (*Dalbergia sisso* L.) collected from the local market. These were air-dried and pyrolyzed at 350 °C in a muffle furnace, then grounded and was passed through a sieve of 200 µm before use. Thermal desorption was done by soil pyrolysis. Soil samples were taken in pre-weighed crucibles and were pyrolyzed at 500 °C in muffle furnace with 60 min residence time^18^.

Earthen pots were filled with 10 kg soil and were maintained at 20% humidity by distilled water^39^. The soil was incubated for a period of 120 days before sowing. Two varieties of lettuce (V1 = Icerberg; V2 = Boston) were grown in control soil and soil contaminated with 10% and 20% crude oil. Three kinds of treatment methods were tested; Combined application of pyrochar and thermal desorption , pyrochar alone and thermal desorption alone under control soil and soil contaminated with 10% and 20% crude oil. The list of test conditions have been mentioned in Table 3.

**Table 3:** Details of treatments and their abbreviation.

Lettuce varieties/germplasm	
V1	Lettuce variety iceberg)
V2	Lettuce variety boston

Treatments	
T0	Control soil (Uncontaminated soil)
T1	10% crude oil contaminated soil
T2	20% crude oil contaminated soil
T3	Pyrochar Treated control soil
T4	Pyrochar + 10% crude oil contaminated soil
T5	Pyrochar + 20% crude oil contaminated
T6	Thermal desorption control soil
T7	Thermal desorption + 10% crude oil contaminated soil
T8	Thermal desorption + 20% crude oil contaminated soil
T9	Combined application of pyrochar and thermal desorption treated control soil
T10	Combined application of pyrochar and thermal desorption treated + 10% crude oil contaminated soil
T11	Combined application of pyrochar and thermal desorption treated + 20% crude oil contaminated soil

### Soil analysis

Soil samples from all treatments were tested for water holding capacity^40^. Saturated soil paste was used to measure pH and electrical conductivity^41^. Soil organic carbon was measured by the oxidation method of Walkley and Black^42^. The sodium acetate method was used to measure cation exchange capacity^43^. Soil texture**,** macro and micronutrients alaysis^44^, available nutrients analysis^45^ was also done.

### Determination of biodegradation percentage

The percentage of oil degradation was calculated using the following formula:

$$ {\text{Biodegradation}}\left( \% \right) \, = {\text{TC}} - {\text{TT}}/{\text{TC}} \times {1}00 $$

whereas TPH is the total petroleum hydrocarbon, TC is the TPH in the control, and TT is the TPH in the treatment.

### Determination of hydrocarbons content of soil samples by GC–MS

Total Petroleum Hydrocarbons (TPH) of all soil samples were determined by EPA method 8015C (USEPA) with slight variation^24^. Briefly, extraction was done by using dichloromethane with the ultrasonication method and analyzed gravimetrically. The extracts were transferred into tare vials nearest 0.0001 g and expressed as g TPH kg^-1^ dry soil. Organic compounds (i.e., C10 to C28) in the extracts were quantified by an Agilent 6890 Gas Chromatography coupled with Time-of-flight Mass Spectrometry and equipped with silica capillary column.

### Potential of remediation techniques for germination attributes of the lettuce plant

A germination experiment was performed in the physiology lab of PMAS Arid Agriculture University, Rawalpindi. Seeds of two lettuce varieties (Iceberg and Boston) obtained from the National Agricultural Research Centre Islamabad, were surface sterilized by treating with sodium hypochlorite (1%) solution for 5 min^46^. After that, seeds were successively washed with distilled water and were placed in Petri plates. There was a total of eleven treatments in this experiment which are described in Supplementary Table 1. Germination percentage, seedling vigor index, and promptness index^46^ were measured for each treatment.

### Potential of remediation techniques for growth attributes of the Lettuce plant

A pot experiment was conducted in the greenhouse of the Botany Department, PMAS- AAUR, Rawalpindi. A complete randomized design (CRD) was applied with three replications. Treatments were the same as described in the above section. Seven sterilized seeds of each variety were sown per pot which was later thinned. Plants were harvested after 45 days of sowing. Fresh and dry biomass was recorded. Leaf area was measured with the help of a leaf area meter. All the samples were collected in zipper bags and stored at − 20 °C freezer for further biochemical assays. In morphological parameters root length, shoot length, shoot fresh/dry weight, root fresh/dry weight, and leaf area were analysed.

### Physiological analysis

Lettuce leaves were analyzed for water potential, using the pressure chamber according to the protocol of Scholander^47^. Excised leaves were inserted in the specimen holder of the pressure vessel of the instrument. The pressure was gradually built up within the pressure vessel until the sap started to ooze out from the exposed cut end of the leaf. Reading at this point corresponded to the negative force with which the water was held within that leaf sample. It was noted and expressed in—MPa.

The osmotic potential was determined by opting for the procedure of Capell and Doerffling^48^. Leaves from each treatment were placed in a 3 ml plastic syringe and stored at − 20 °C freezer. After a few days, when the leaves became frozen, these syringes were taken out and pressed to collect the leaf sap from the thawed samples in Eppendorf tubes. The10 µL from each sample was taken readings were obtained by vapor pressure osmometer (VIESCOR 5520 VX R) in mmol/kg and these values were converted in to (-MPA) with the help of this formula

$$ {\text{Osmotic }}\;{\text{potential }} = {\text{ Osmolality}}\; \, \left( {{\text{mmol}}} \right) \, \times \, 0.{831} \times { 1}0^{{ - {5}}} {\text{T }}\left( {^\circ {\text{K}}} \right) $$

where T is temperature expressed in °K.

For determining membrane stability index (MSI), Premchandra et al.^49^, method which, was used. For this purpose, 100 mg leaf discs were carefully washed first with tap water and then with double distilled water. After washing, leaf discs were heated in 10 mL double distilled water at 40 °C in a water bath for 30 min. After 30 min, electrical conductivity (C_1_) of this sample was noted using the EC meter. After noting the first EC reading the same sample was placed in a water bath at 100 °C for 10 min. and after this process, electrical conductivity (C_2_) was again recorded. For calculating the Membrane stability index following equation was used

$$ {\text{MSI }} = \, \left( {{1} - \frac{C1}{{C2}}} \right) \, \times { 1}00 $$

Arnon^50^ method was opted for estimating chlorophyll and carotenoid content of leaf samples. Leaves were weighed and then crushed in a clean pestle and mortar. 5 mL of 80% acetone was added to each crushed sample. This sample was then centrifuged, and the supernatant was taken into the cuvette of the spectrophotometer. The absorbance of the extract was recorded at different wavelengths i.e., 663 nm and 645 nm and 470 nm. The values of chlorophyll a, b, total chlorophyll, and carotenoid were calculated by following formulae given by Lichtenthaler and Welburn^51^

$$ {\text{Chlorophyll }}\;a \, (\mu {\text{g}}/{\text{mL}}) \, = {12}.{21 }\left( {{\text{A}}_{{{663}}} } \right) \, - { 2}.{81 }\left( {{\text{A}}_{{{645}}} } \right) $$

$$ {\text{Chlorophyll }}\;b \, (\mu {\text{g}}/{\text{mL}}) \, = { 2}0.{13 }\left( {{\text{A}}_{{{645}}} } \right) \, {-}{ 5}.0{3 }\left( {{\text{A}}_{{{663}}} } \right) $$

$$ {\text{Total }}\;{\text{chlorophyll }}\;(\mu {\text{g}}/{\text{mL}}) \, = { 2}0.{2 }\left( {{\text{A}}_{{{645}}} } \right) \, + { 8}.0{2 }\left( {{\text{A}}_{{{663}}} } \right) $$

$$ {\text{Carotenoid }}\;{\text{content }}(\mu {\text{g}}/{\text{mL}}) \, = \, \left[ {{1}000{\text{A}}_{{{47}0}} - { 3}.{27 }\left( {{\text{chlorophyll }}\;a} \right) \, {-}{ 1}0{4 }\left( {{\text{chlorophyll }}\;b} \right)} \right] \, /{ 227} $$

### Biochemical analysis

Proline was measured following the method of Bates et al.^52^. Leaf material (0.1 g) was homogenized with 4 mL sulfosalicylic acid (3.0%) with the help of pestle and mortar. This sample was previously kept overnight at 5 °C. The suspension was centrifuged at room temperature at 3000 rpm for 5 min; Supernatant was mixed with 4 mL acetic ninhydrin reagent and mechanically shaken and the contents in the tubes were heated for one hour at 100 °C. After cooling the content in the tubes and this solution was extracted with 4 mL of toluene with the help of a separating funnel. Then the toluene layer was separated and its absorbance was recorded with a spectrophotometer at 520 nm. The concentration of the proline of these unknown samples was calculated by using the standard curve of proline.

Soluble protein was determined following the method of Lowry et al.^53^, using Bovine Serum Albumin (BSA) as standard. Fresh leaves (0.1 g) were ground in 1 mL of sodium phosphate buffer (pH 7.5) with the help of mortar and pestle and this mixture was centrifuged at 3000 rpm for 10 min. The supernatant (0.1 mL) was taken in the test tubes. Total volume was made 1 mL with the help of Distilled water. 1mLof reagent made by mixing 50 mL of solution A (2.0 g Na_2_CO_3_) 0.4 g NaOH (0.1 N) and 1 g Na–K tartrate was dissolved in 100 mL of distilled water) and 1 mL of solution B (CuSO_4_.5H_2_O (0.5 g) dissolved in 100 mL of distilled water) was added. After shaking for 10 min. 0.1 mL of Folin phenol reagent (diluted with distilled water in the ratio 1:1) was added. After 30 min absorbance of each sample was recorded at 650 nm after 30 min.

For the quantification of free amino acids, the leaf sample (each 0.5 g) was ground in 10 mL of potassium phosphate buffer. The mixture was centrifuged at 12,000×*g* at 4 °C. To 1 mL of the supernatant, 1 mL of ninhydrin (2%) and 1 mL of pyridine (10%) were added, and the mixture was placed in a water bath for 30 min. After cooling all samples, absorbance was read at 750 nm using a spectrophotometer^54^.

Dubois et al.^55^, the method was used for estimating the sugar content of leaves. Leaf material (0.5 g) was crushed with the help of a clean pestle and mortar, 10 mL of distilled water was added to this material. Then this homogenized material was filtered. 0.1 mL of the filtrate from each sample was taken in the separate test tube and 1 mL of (5% v/v), phenol was added and left at room temperature for one hour. After 1 h 5 mL of H_2_SO_4_ was added to the test tubes. Then absorbance of each sample was noted at 420 nm by a spectrophotometer. The standard curve of glucose solution of known concentration was used to estimate the sugar content of the samples.

Enzyme extract was prepared by grinding one gram of leaf in liquid nitrogen. The obtained powder was added in 10 mL of 50 mM phosphate buffer (pH 7.0) and was mixed with 1 mM Ethylene Diamine Tetra Acetic acid (EDTA) and 1% polyvinylpyrrolidone (PVP). The whole mixture was centrifuged at 13,000×*g* for 20 min at 4 °C. The supernatant was used for the enzyme assay.

The catalase (CAT) content was estimated by observing the degradation of H_2_O_2_ at 240 nm^56^. Catalase activity (U mg protein^−1^) was calculated from the molar absorption coefficient of 40 mm^−1^ cm^−1^ for H_2_O_2_. Peroxidase dismutase (POD) was determined by following the procedure of Rao^57^. The reaction mixture consisted of 10 μL of crude enzyme extract, 20 μL of 100 mM guaiacol, 10 μL of 100 mM H_2_O_2_, and 160 μL of 50 mM sodium acetate (pH 5.0). Absorbance was recorded at 450 nm.

Superoxide dismutase (SOD) activity was determined by the method of Beauchamp and Fridovich^58^. The reaction mixture (3 mL) was composed of 13 mM methionine, 0.075 mM NBT, 0.1 mM EDTA, 0.002 mM riboflavin, and 0.1 mL of enzyme extract in 50 mM phosphate buffer (pH 7.8). The mixture in the tube was placed under fluorescent light for 15 min thereafter the reaction was stopped by turning the lights off. A complete reaction mixture (non-irradiated) was used as a blank. The absorbance was read at 560 nm with a spectrophotometer. One unit of SOD activity was defined as the amount of enzyme, which reduced the absorbance reading by 50% as compared to the control (lacking enzyme).

### Statistical analysis

The morphological, physiological, and biochemical parameters were calculated, and the data were subjected to the analysis of variance (ANOVA) by using statistics 8.1 software. Mean values were compared by the least significant difference (LSD) at P ≤ 0.05^59^ Heatmap for correlation coefficient was generated using web tool clustvis (https://biit.cs.ut.ee/clustvis/).

## Supplementary Information

Supplementary Information.
