# Supplementary material for: Comparison of plant growth and remediation potential of pyrochar and thermal desorption for crude oil-contaminated soils
Source: Sci Rep. 2021 Feb 2;11:2817. doi: 10.1038/s41598-021-82243-y (PMC7854674; doi:10.1038/s41598-021-82243-y)
Supplement: Supplementary file 1 — Supplementary Information. [file 41598_2021_82243_MOESM1_ESM.docx]

**Comparison of plant growth and remediation potential of pyrochar and thermal desorption for crude oil-contaminated soils**

Noshin Ilyas, Uzma Shoukat, Maimoona Saeed, Nosheen Akhtar, Humaira Yasmin, Wajiha Khan, Sumera Iqbal

**Supplementary Table S1** Physiochemical properties of hydrocarbon contaminated, biochar and thermal remediated soil.

| **Treatments** | **pH** | **EC(dSm^-1^)** | **Soil Texture** | **OC (%)** | **Available nutrients**  **(%)** | | | **SMC (%)** |
| --- | --- | --- | --- | --- | --- | --- | --- | --- |
|  |  |  |  |  | **N** | **P** | **K** |  |
| T0 | 6.99 | 0.72 | Sandy clay loam | 0.72 | 0.44 | 2 | 0.5 | 11.26 |
| T1 | 6.75 | 0.65 | Sandy clay loam | 1.54 | 0.31 | 1.86 | Traces | 8.5 |
| T2 | 6.44 | 0.56 | Sandy clay loam | 3.97 | 0.24 | 1.70 | Traces | 7.1 |
| T3 | 7.01 | 0.79 | Sandy clay loam | 0.92 | 0.56 | 2.15 | 0.830 | 11.5 |
| T4 | 6.80 | 0.69 | Sandy clay loam | 1.62 | 0.49 | 1.90 | 0.62 | 9 |
| T5 | 6.5 | 0.60 | Sandy clay loam | 4.2 | 0.29 | 1.79 | 0.57 | 7.7 |
| T6 | 7.0 | 0.72 | Sandy clay loam | 0.73 | 0.45 | 2.05 | 0.5 | 11.3 |
| T7 | 6.76 | 0.64 | Sandy clay loam | 1.53 | 0.32 | 1.87 | Traces | 6.7 |
| T8 | 6.65 | 0.55 | Sandy clay loam | 3.95 | 0.25 | 1.71 | Traces | 4.3 |
| T9 | 6.97 | 0.70 | Sandy clay loam | 1.2 | 0.58 | 2.16 | 0.7 | 11.8 |
| T10 | 6.73 | 0.62 | Sandy clay loam | 1.8 | 0.52 | 1.921 | 0.65 | 9.5 |
| T11 | 6.43 | 0.52 | Sandy clay loam | 4.8 | 0.36 | 1.80 | 0.5 | 9.2 |

Where, T0= control soil, T1= 10% crude oil contaminated soil, T2= 20% crude oil contaminated soil, T3= Biochar treated control soil, T4= Biochar + 10% crude oil contaminated soil, T5= Biochar + 20% crude oil contaminated soil, T6= Thermal decontaminated control soil, T7= Thermal decontamination+10% crude oil contaminated soil, T8= Thermal remediation + 20% crude oil contaminated soil, T9= Biochar + Thermal decontaminated control soil, T10= Biochar + Thermal decontamination +10% crude oil contaminated soil, T11= Biochar + Thermal decontamination + 20% crude oil contaminated. soil V1= Iceberg, V2= Boston.

**Supplementary Figure 1.** Germination percentage, Seedling Vigor Index and Promptness Index of two lettuce varieties growing in biochar and thermal remediated soil.

This data displays the means and standard deviation (n=3). Different letters show significant differences (p<0.05).

Detail of treatments: Where, T0= control soil, T1= 10% crude oil contaminated soil, T2= 20% crude oil contaminated soil, T3= Biochar treated control soil, T4= Biochar + 10% crude oil contaminated soil, T5= Biochar + 20% crude oil contaminated soil, T6= Thermal decontaminated control soil, T7= Thermal decontamination+10% crude oil contaminated soil, T8= Thermal remediation + 20% crude oil contaminated soil, T9= Biochar + Thermal decontaminated control soil, T10= Biochar + Thermal decontamination +10% crude oil contaminated soil, T11= Biochar + Thermal decontamination + 20% crude oil contaminated. soil V1= Iceberg, V2= Boston.
